# WHOM WOULD YOU HIRE? AGEISM IN ORGANIZATIONAL CONTEXTS

**DOI:** 10.1093/geroni/igz038.2112

**Published:** 2019-11-08

**Authors:** Hannah Swift, Vanessa Dias, Dominic Abrams

**Affiliations:** University of Kent, Canterbury, England, United Kingdom

## Abstract

People want to work at older ages, yet ageism and discrimination remain a barrier. Using theories of prejudice, social role theory, and conceptual models of age diversity in organisational contexts, we explore age-bias in hiring practices (Study 1) and how to reduce it (Study 2). Study 1 (N=150) investigated pro-youth bias in hiring practices and how this manifests depending on job/occupation. Study 2 (N=150) investigated whether pro-youth bias is reduced by manipulating organisational culture. In both studies, participants were given a fictional organisation, a job ad, and two applicants’ profiles manipulated to represent men one each in their 30s and 50s. Study 1 supported the social role theory hypothesis: people match candidates to the age profile of the job. In the age-neutral job occupation participants chose equally between candidates. Study 2 supported the hypothesis that pro-youth bias can be mitigated when age-diverse nature of the organisational culture is made salient.

